# Liver Fibrosis Scores and Clinical Outcomes in Patients With COVID-19

**DOI:** 10.3389/fmed.2022.829423

**Published:** 2022-04-08

**Authors:** Jing Zhang, Fuwei Liu, Tiangang Song, Zhangwang Li, Panpan Xia, Xiaoyi Tang, Minxuan Xu, Yunfeng Shen, Jianyong Ma, Xiao Liu, Peng Yu

**Affiliations:** ^1^Department of Anesthesiology, The Second Affiliated Hospital of Nanchang University, Nanchang, China; ^2^Department of Cardiology, The Affiliated Ganzhou Hospital of Nanchang University, Ganzhou, China; ^3^The Second Clinical Medical College of Nanchang University, Nanchang, China; ^4^Department of Metabolism and Endocrinology, The Second Affiliated Hospital of Nanchang University, Nanchang, China; ^5^Institute for the Study of Endocrinology and Metabolism in Jiangxi Province, Nanchang, China; ^6^Department of Pharmacology and Systems Physiology, University of Cincinnati College of Medicine, Cincinnati, OH, United States

**Keywords:** COVID-19, liver fibrosis scores, discharge, mortality, prognosis

## Abstract

**Background and Aims:**

We investigated the association between liver fibrosis scores and clinical outcomes in patients with COVID-19.

**Methods:**

We performed a *post-hoc* analysis among patients with COVID-19 from the trial study Outcomes Related to COVID-19 treated with Hydroxychloroquine among Inpatients with symptomatic Disease (ORCHID) trial. The relationship between aspartate aminotransferase (AST) to platelet ratio index (APRI), non-alcoholic fatty liver disease fibrosis score (NFS), Fibrosis-4 index (FIB-4), and discharge and death during the 28-days of hospitalization was investigated.

**Results:**

During the 28 days after randomization, 237 (80.6%) patients were discharged while 31 (10.5%) died among the 294 patients with COVID-19. The prevalence for advanced fibrosis was estimated to be 34, 21.8, and 37.8% for FIB-4 (>2.67), APRI (>1), and NFS (>0.676), respectively. In multivariate analysis, FIB-4 >2.67 [28-days discharge: hazard ratio (HR): 0.62; 95% CI: 0.46–0.84; 28-days mortality: HR: 5.13; 95% CI: 2.18–12.07], APRI >1 (28-days discharge: HR: 0.62; 95% CI: 0.44–0.87; 28-days mortality: HR: 2.85, 95% CI: 1.35–6.03), and NFS >0.676 (28-days discharge: HR: 0.5; 95% CI: 0.35–0.69; 28-days mortality: HR: 4.17; 95% CI: 1.62–10.72) was found to significantly reduce the discharge rate and increase the risk of death. Additionally, FIB-4, APRI, and NFS were found to have good predictive ability and calibration performance for 28-day death (C-index: 0.74 for FIB-4, 0.657 for APRI, and 0.745 for NFS) and discharge (C-index: 0.649 for FIB-4, 0.605 for APRI, and 0.685 for NFS).

**Conclusion:**

In hospitalized patients with COVID-19, FIB-4, APRI, and NFS may be good predictors for death and discharge within 28 days. The link between liver fibrosis and the natural history of COVID-19 should be further investigated.

## Introduction

Coronavirus disease 2019 (COVID-19 is an infectious disease caused by a novel beta coronavirus belonging to the sarbecovirus subgenus of the Coronavirus family, namely respiratory syndrome coronavirus 2 (SARS-CoV-2) (1). COVID-19 can cause severe multisystem complications and has led to over 4 million deaths worldwide as of 29 June 2021 (2). COVID-19 is more than respiratory disease, it is a systemic infection that affects the gastrointestinal tract, heart, cardiovascular system, kidneys, and other organs (3).

Increasing evidence has suggested that liver injury is one of the most common complications of COVID-19 (1) due to the hepatotropism of SARS-CoV-2 and has been significantly associated with an increased risk of developing severe COVID-19 disease and death (2, 3). Furthermore, patients with chronic liver diseases, such as non-alcoholic fatty liver disease (NAFLD), were more prone to develop liver injury after COVID-19 infection (4). Advanced liver fibrosis is the primary determinant of progression from less malignant liver diseases to cirrhosis and liver failure. In recent years, several simple non-invasive indices (e.g., Fibrosis-4 index [FIB-4], AST to platelet ratio index [APRI], NAFLD fibrosis score [NFS]) were developed to distinguish the severity of fibrosis. These indices have good performance and have shown to be strong predictors for all-cause death in the general population or individuals with chronic liver diseases (4–8). Furthermore, markers of liver fibrosis scores, such as aspartate aminotransferase (AST) and alanine aminotransferase ratio (ALT), were significantly associated with adverse outcomes in patients with COVID-19 (2, 6). However, the association between FIB-4, APRI, or NFS and hospital discharge or death in patients with COVID-19 is unknown. Therefore, we performed a *post-hoc* analysis to determine the association of liver fibrosis scores on clinical outcomes in patients with COVID-19 based on the dataset from Outcomes Related to COVID-19 Treated With Hydroxychloroquine Among Inpatients With Symptomatic Disease (ORCHID) trial, which is a blinded, placebo-controlled randomized trial conducted across 34 hospitals in the US.

## Methods

The reporting of the research followed the Strengthening the Reporting of Observational Studies in Epidemiology (STROBE) statement (9).

### Data Source

This is a *post-hoc* analysis of the ORCHID trial (10). The main results of this trial have been published (11). Briefly, ORCHID is a multicenter, blinded, randomized clinical trial that compared hydroxychloroquine with placebo on the clinical status of hospitalized patients with moderate and severe COVID-19. The trial included 479 hospitalized patients with COVID-19 across 43 hospitals in the US between 2 April 2020 and 19 June 2020. The inclusion criteria were as follows: (1) adults hospitalized with COVID-19 <48 h with laboratory-confirmed SARS-CoV-2 positivity; (2) symptoms of respiratory illness for <10 days. The main exclusion criteria were treatment with hydroxychloroquine or chloroquine or medications that prolong the QTC interval to > 500 ms within 10 days of hospitalization. The primary outcome was clinical status during 14 days after randomization as assessed with the 7-category ordinal scale (the COVID Outcomes Scale) recommended by the WHO. The second outcome was the COVID Outcomes Scale and the clinical outcomes [including 14- and 28-day death, an Extra Corporeal Membrane Oxygenation (ECMO) event, or intensive care unit (ICU) admission]. Patients were followed up for death until 28 days, following hydroxychloroquine randomization using in-hospital records and telephone follow-up after discharge. The Prevention and Early Treatment of Acute Lung Injury Clinical Trials Network Clinical Coordinating Center reviewed all the information to ensure data quality. A central institutional review board at Vanderbilt University Medical Center approved the ORCHID. Informed consent for participation was obtained from the patients or legally authorized representatives. Notably, the investigators of the randomized controlled trials (RCTs) were not involved in this study. After excluding patients with lost to follow-up and missing baseline characteristics, finally, 294 patients were included in this study for FIB-4 and APRI. Specifically, in the assessment of NFS, there were 262 patients included.

### Assessment of Liver Fibrosis

Liver fibrosis scores were computed using the following formulas (12, 13):

Fibrosis-4: [age (years) × AST (U/L)]/[platelet (×10^9^/L) × ALT (U/L)].

NAFLD fibrosis score: −1.675+0.037 × age (years) +0.094 × body mass index (BMI) (kg/m^2^) +1.13 × diabetes (yes = 1, no = 0) +0.99 × AST [U/L]/ALT [U/L]−0.013 × platelet (10^9^/L)−0.66 × albumin (g/dL).

APRI: [AST (U/L)/upper limit of normal^*^100]/platelet (×10^9^/L) ratio.

To be specific, in order to minimize the overestimation of predicted advanced fibrosis, patients belonging to the intermediate above scores category were considered negative control (reference). Therefore, the following cutoffs were used: FIB-4 (2.67), APRI (1), and NFS (0.676).

### Outcomes

The outcomes were defined as 28-days all-cause death and 28-days discharge through post-randomization. The detailed definitions of these outcomes can be found in the previous report (11).

### Covariates

Potential confounders at baseline were collected, including demographics (age, sex, and race), comorbidities (BMI, hypertension, diabetes, chronic kidney disease, coronary artery disease, and chronic obstructive pulmonary disease), laboratory measurements (white blood cell count, platelet count, creatinine, AST, and ALT), duration of symptoms at baseline, Sequential Organ Failure Assessment (SOFA) score at enrollment, symptoms of acute respiratory infection (shortness of breath, cough, and fever), chronic medication history (angiotensin-converting-enzyme inhibitor, angiotensin II receptor blocker, and corticosteroids), and inpatient treatments (e.g., corticosteroid, tocilizumab, and azithromycin).

Chronic liver disease includes chronic hepatitis without portal hypertension and cirrhosis with portal hypertension or variceal bleeding.

### Statistical Analysis

Continuous variables were expressed as the means with *SD*s (normal distribution) or medians with interquartile ranges (IQRs; non-normal distribution). The differences between groups for continuous variables were compared using the unpaired Student's *t*-test (normal distribution) or Wilcoxon-Mann-Whitney tests (non-normal distribution). Categorical variables, which were reported as counts and percentages, were compared between groups using the χ^2^ test. For non-normally distributed categorical variables, the Kruskal-Wallis test was used. Survival analysis was performed using Kaplan–Meier estimates tested by the log-rank method. Cox proportional hazards models were used to calculate the adjusted risk estimates [i.e., hazard ratios (HRs) and their CIs]. The selection of adjusted covariates in the multivariable models was based on the backward stepwise method with a significance level of <0.1, including all the baseline factors. We evaluated the discriminatory abilities of the liver fibrosis scores for predicting outcomes using the C-index calculated by the area under the receiver operating characteristic curve. For internal model and score validation, we used k-fold in 10-fold cross-validation with repeating 200 times in the original sample. All statistical analyses were performed using SPSS Statistics version 26 (IBM Corp., Armonk, N.Y., USA) and R version 4.0.3 software (The R development Core Team). A two-sided *p*-value < 0.05 was considered statistically significant.

### Supplementary Analysis

To evaluate the robustness of the findings, we conducted a sensitivity analysis by (1) extending the definition of 28-day death to in-hospital death; (2) excluding the patients with pre-existing chronic liver diseases at baseline; (3) using a competing model, in which the death was defined as a competing event.

## Results

### Baseline Characteristics

Among the 294 hospitalized patients with respiratory illness from COVID-19, 2.4% (7/294) had chronic liver diseases, which included chronic hepatitis and cirrhosis. Advanced liver fibrosis assessed using FIB-4, APRI, and NFS was 34, 21.8, and 37.8%, respectively. The mean age was 56.5 years (*SD*: 15.6), with a lower proportion of women (40.1%). The most frequent comorbidities were hypertension (51.7%), diabetes mellitus (36.4%), and obesity (23%). The baseline characteristics of the patients with COVID-19 grouped by FIB-4, APRI, and NFS are shown in Table 1.

**Table 1:** Baseline characteristics of included patients stratified by FIB-4, APRI, and NFS.

**Characteristics**	**Total‡**	**FIB-4**		**APRI**		**Total^§^**	**NFS**	
	**(***n*** = 294)**	**≤2.67**	**>2.67**	**≤1.0**	**>1.0**	**(***n*** = 262)**	**≤0.676**	**>0.676**
		**(***n*** = 194)**	**(***n*** = 100)**	**(***n*** = 230)**	**(***n*** = 64)**		**(***n*** =163)**	**(***n*** =99)**
Age, years	56.5 ± 15.6	52.10 ± 14.5	64.95 ± 14.1^*^	56.00 ± 16.1	59.50 ± 14.6	56.16 ± 15.5	53.36 ± 15.4	60.77 ± 14.5^*^
Sex, male	176 (59.9)	116 (59.8)	60 (60.0)	132 (57.4)	44 (68.8)	155 (59.2)	102 (62.6)	53 (53.5)
**Race**								
White or Caucasian	127 (43.2)	81 (41.8)	46 (46.0)	93 (40.4)	34 (53.1)	112 (42.7)	69 (42.3)	43 (43.4)
Black or African American	74 (25.2)	46 (23.7)	28 (28.0)	61 (26.5)	13 (20.3)	70 (26.7)	36 (22.1)	34 (34.3)^*^
Others	97 (23.0)	70 (36.1)^*^	27 (27.0)	79 (34.3)	18 (28.1)	83 (31.7)	59 (36.2)	27 (24.3)^*^
BMI, kg/m^2^	31.1 (26.8, 37.1)	31.2 (7.1, 38.4)	29.4 (26.5, 35.9)	31.1 (27.1, 37.7)	30.1 (25.8, 36.0)	31.1 (27.1, 37.4)	29.4 (25.9, 29.4)	34.5 (29.2, 43.1)^*^
Scores, FIB-4	1.89 (1.19, 3.45)	1.46 (1.01, 1.89)	4.43 (3.41, 6.56)^*^	0.42 (0.26, 0.60)	1.66 (1.20, 2.81)^*^	0.06 (−1.18, 1.53)	−0.86 (−1.80, −0.17)	1.96 (1.19, 2.88)^*^
**Chronic health conditions**								
Peripheral vascular disease	12 (4.1)	7 (3.6)	5 (5.0)	10 (4.3)	2 (3.1)	10 (3.8)	5 (3.1)	5 (5.1)
COPD	26 (8.8)	15 (7.7)	11 (11.0)	20 (8.7)	6 (9.4)	21 (8.0)	10 (6.1)	11 (11.1)
Hypertension	152 (51.7)	91 (46.9)	61 (61.0)^*^	116 (50.4)	36 (56.3)	135 (51.5)	74 (45.4)	61 (61.6)^*^
Coronary artery disease	21 (7.1)	8 (4.1)	13 (13.0)^*^	16 (7.0)	5 (7.8)	19 (7.3)	6 (3.7)	143 (13.1)^*^
Diabetes mellitus	107 (36.4)	65 (33.5)	42 (42.0)	83 (36.1)	24 (37.5)	94 (35.9)	34 (20.9)	60 (60.6)^*^
Moderate to severe kidney disease^†^	27 (9.2)	11 (5.7)	16 (16.0)^*^	19 (8.3)	8 (12.5)	22 (8.4)	6 (3.7)	16 (16.2)^*^
Congestive heart failure	25 (8.5)	10 (5.2)	15 (15.0)^*^	19 (8.3)	6 (9.4)	22 (8.4)	6 (3.7)	16 (16.2)^*^
Prior myocardial infarction	15 (5.1)	9 (4.6)	6 (6.0)	12 (5.2)	3 (4.7)	15 (5.7)	12 (7.4)	3 (3.0)
Cerebrovascular disease	17 (5.8)	8 (4.1)	9 (9.0)	12 (5.2)	5 (7.8)	14 (5.3)	6 (3.7)	8 (8.1)
Liver diseases	7 (2.4)	4 (2.1)	3 (3.0)	6 (2.6)	1 (1.6)	6 (2.3)	2 (1.2)	4 (4.0)
**Home medication**								
Corticosteroids	32 (10.9)	16 (8.2)	16 (16.0)^*^	22 (9.6)	10 (15.6)	30 (11.5)	19 (11.7)	11 (11.1)
ACE inhibitors	44 (15.0)	26 (13.4)	18 (18.0)	33 (14.3)	11 (17.2)	41 (15.6)	21 (12.9)	20 (20.2)
ARB	28 (9.5)	17 (8.8)	11 (11.0)	20 (8.7)	8 (12.5)	27 (10.3)	16 (9.8)	11 (11.1)
NSAIDs	41 (13.9)	26 (13.4)	15 (15.0)	21 (13.9)	9 (14.1)	34 (13.0)	19 (11.7)	15 (15.2)
**Symptoms of acute respiratory infection**								
Cough	171 (58.2)	118 (60.8)	53 (53.0)	135 (58.7)	36 (56.3)	154 (18.8)	96 (58.9)	58 (58.6)
Fever	170 (57.8)	108 (55.7)	62 (62.0)	133 (57.8)	37 (57.8)	151 (57.6)	90 (55.2)	61 (61.6)
Shortness of breath	216 (73.5)	147 (75.8)	69 (69.0)	168 (73.0)	48 (75.0)	196 (74.8)	121 (74.2)	75 (75.8)
Sore throat	20 (6.8)	15 (7.7)	5 (5.0)	17 (7.4)	4 (4.7)	16 (6.1)	11 (6.7)	5 (5.1)
**Pre-medication up to randomization**								
Hydroxychloroquine	2 (0.7)	2 (1.0)	0 (0.0)	2 (0.9)	0 (0.0)	2 (0.8)	2 (1.2)	0 (0.0)
Remdesivir	18 (6.1)	11 (5.7)	7 (7.0)	15 (6.5)	3 (4.7)	16 (6.1)	8 (4.9)	8 (8.1)
Lopinavir/ritonavir	0 (0.0)	0 (0.0)	0 (0.0)	0 (0.0)	0 (0.0)	0 (0.0)	0 (0.0)	0 (0.0)
Corticosteroids	27 (9.2)	18 (9.3)	9 (9.0)	22 (9.6)	5 (7.8)	23 (8.8)	19 (11.7)	4 (4.0)^*^
Azithromycin	94 (32.0)	59 (30.4)	35 (35.0)	69 (30.0)	25 (39.1)	86 (32.8)	47 (28.8)	39 (39.4)
Chloroquine	0 (0.0)	0 (0.0)	0 (0.0)	0 (0.0)	0 (0.0)	0 (0.0)	0 (0.0)	0 (0.0)
**In-hospital medication**								
Corticosteroids	67 (22.8)	39 (20.1)	28 (28.0)	49 (21.3)	18 (28.1)	62 (23.7)	32 (19.6)	30 (30.3)^*^
Tocilizumab	21 (7.1)	11 (5.7)	10 (10.0)	13 (5.7)	8 (12.5)	21 (8.0)	10 (6.1)	11 (11.1)
Sarilumab	0 (0.0)	0 (0.0)	0 (0.0)	0 (0.0)	0 (0.0)	0 (0.0)	0 (0.0)	0 (0.0)
Interferon	0 (0.0)	0 (0.0)	0 (0.0)	0 (0.0)	0 (0.0)	0 (0.0)	0 (0.0)	0 (0.0)
Immunomodulating medication	4 (1.4)	2 (1.0)	2 (2.0)	3 (1.3)	1 (1.6)	4 (1.5)	2 (1.2)	2 (2.0)
Bilateral opacities/infiltrates	192 (65.3)	123 (63.4)	69 (99.0)	146 (66.7)	46 (73.0)	172 (65.6)	107 (69.0)	65 (68.4)
**Lab test**								
Respiratory SOFA score	2.00 (1.00, 2.00)	1.00 (1.00, 2.00)	2.00 (1.00, 3.00)^*^	1.00 (1.00, 2.00)	2.00 (1.00, 3.00)^*^	2.00 (1.00, 2.00)	1.00 (1.00, 2.00)	2.00 (1.00, 3.00)
Systolic blood pressure, mmHg	109.0 (99.0, 123.0)	110.5 (101.3, 123.8)	106.0 (96.0, 120.0)	110.0 (100.0, 123.0)	105.5 (93.5, 121.5)	109.0 (99.0, 123.0)	112.0 (102.0, 124.0)	106.0 (95.0, 122.0)^*^
White blood cell count, /mm^3^	6,190 (4,300, 8,008)	6,500 (4,600, 8,450)	5,300 (4,080, 6,700)^*^	6,300 (4,450, 8,300)	5,300 (4,000, 7,050)	6,200 (4,240, 8,175)	6,500 (4,700, 8,700)	5,800 (4,125, 7,450)
Hemoglobin, g/dL	12.9 (11.5, 14.2)	13.0 (11.7, 14.2)	12.8 (11.3, 14.4)	12.9 (11.5, 14.1)	13.1 (11.9, 14.9)	13.0 (11.6, 14.3)	13.2 (12.1, 14.4)	12.5 (10.8, 14.1)^*^
Platelet, /mm3	217.5 (162.8, 270.5)	243.0 (105.0, 199.0)	146.5 (117.3, 199.3)^*^	233.0 (185.8, 286.5)	145.5 (109.3, 202.3)^*^	216.0 (162.0, 272.3)	243.0 (196.0, 311.0)	178.0 (127.0, 219.0)^*^
BUN, mg/dL	14.0 (11.0, 26.0)	13.5 (10.0, 21.0)	19.5 (12.0, 35.8)^*^	14.0 (11.0, 24.0)	17.5 (11.3, 35.0)	14.0 (11.0, 25.0)	13.0 (10.0, 19.0)	21.0 (13.0, 35.0)^*^
AST, U/L	41.5 (28.8, 66.3)	35.0 (25.0, 48.3)	67.0 (42.5, 111.0)^*^	37.0 (27.0, 49.0)	95.0 (73.5, 156.5)^*^	41.0 (29.0, 66.3)	38.0 (27.0, 61.0)	47.0 (34.0, 79.0)^*^
ALT, U/L	30.0 (19.8, 52.5)	27.0 (18.8, 47.3)	38.5 (24.0, 65.0)^*^	25.0 (17.8, 43.0)	62.0 (38.3, 92.8)^*^	30.0 (20.0, 52.5)	31.0 (21.0, 59.0)	28.0 (18.0, 43.0)
ALP, IU/L	75.0 (58.0, 95.0)	74.0 (58.3, 93.0)	76.0 (58.0, 104.0)	75.0 (58.8, 93.0)	77.0 (57.5, 140.0)	75.0 (57.5, 93.0)	75.0 (57.0, 90.0)	74.0 (59.0, 101.5)
ALB, g/dL	3.5 (3.1, 3.9)	3.6 (3.1, 3.9)	3.4 (3.1, 3.8)	3.6 (3.1, 3.9)	3.5 (3.1, 3.8)	3.6 (3.1, 3.9)	3.6 (3.3, 4.0)	3.3 (2.8, 3.7)^*^

*(IQR) for non-normally distributed data, M ± SD for normally distributed data, and n (%) for categoric variables*.

*BMI, body mass index; COPD, chronic obstructive pulmonary disease; ACEI, angiotension converting enzyme inhibitors; ARB, Angiotensin Receptor Blockers; NSAID, non-steroidal anti-inflammatory drugs; SOFA, Sequential Organ Failure Assessment; BUN, blood urea nitrogen; ALT, alamine aminotransferase; AST, aspartate aminotransferase; ALP, alkaline phosphatase; ALB, albumin; FIB-4, Fibrosis-4; APRI, Aspartate Aminotransferase -to-platelet ratio index*.

† *Moderate to severe kidney disease was defined as Cr >3, ESRD, chart diagnosis of CKD stage 5 (eGFR <15 mL/min/1.73m^2^) not on dialysis*.

‡ *Excluding patients with missing values for ALT, AST and PLT*.

§ *Excluding patients with missing values for BMI, ALT, AST, PLT, and ALB*.

* *Represents P < 0.05*.

The associations of individual components for FIB-4 (age, AST, ALT, and platelet), APRI (AST and platelet), and NFS (age, BMI, diabetes, AST, ALT, platelet, and albumin) scores with hospital discharge and death through the 28-days are presented in Supplementary Material S1.

### Association of FIB-4 Scores With Clinical Outcomes in Patients With COVID-19

Individual components of FIB-4 (age, AST, ALT, and platelet) in patients with FIB-4 >2.67 were significantly higher compared to the FIB-4 ≤ 2.67 group. Compared to the FIB-4 ≤ 2.67 group, patients with FIB-4 >2.67 had a higher frequency of hypertension, coronary artery disease, moderate to severe kidney disease, congestive heart failure, higher respiratory SOFA scores, lower white blood cell counts, and more usage of corticosteroids at home (Table 1).

**Figure 1:**
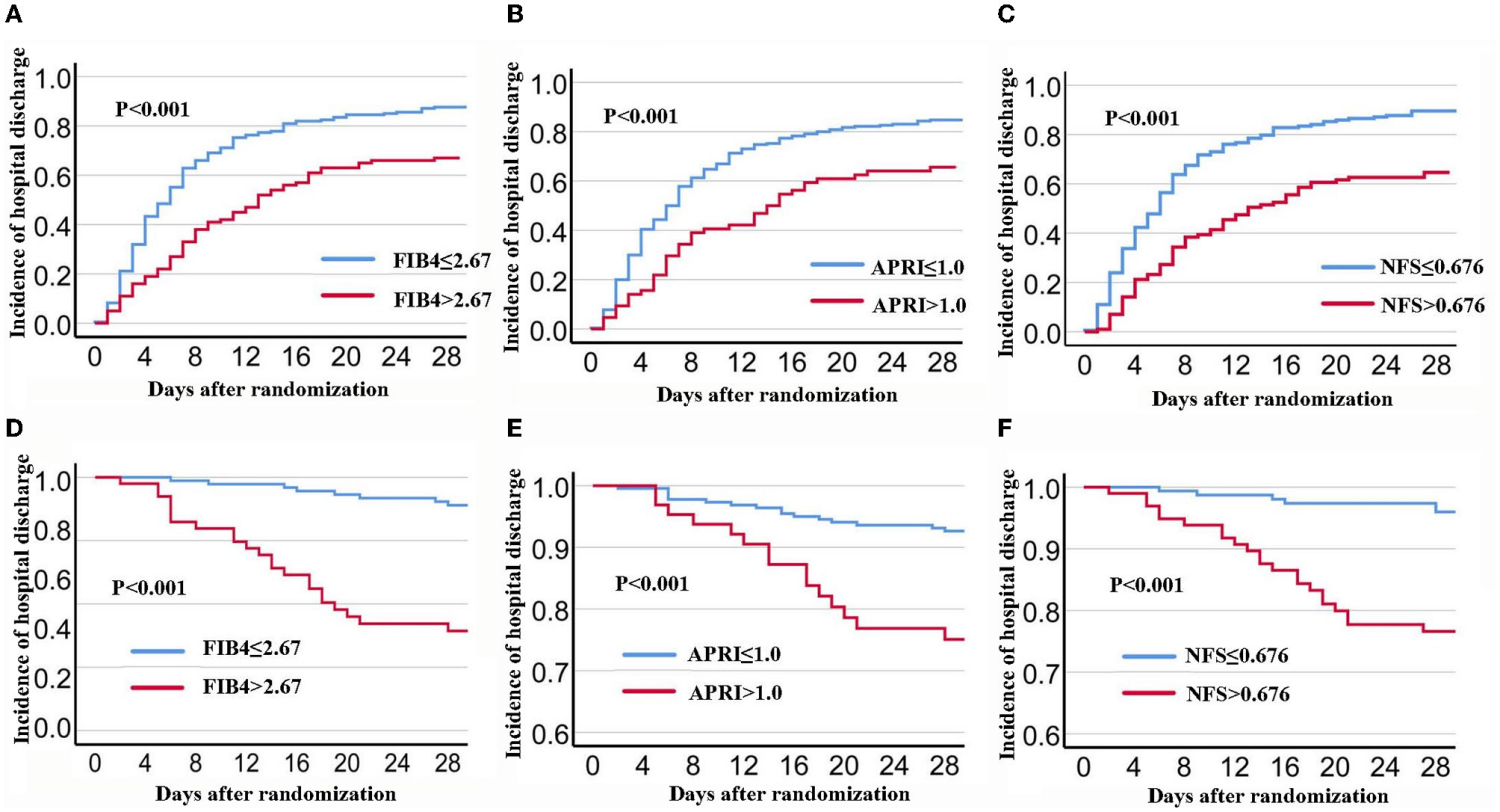
K-M Survival curves for 28-days discharge and 28-days mortality stratified by FIB-4, APRI, and NFS in patients with COVID-19. **(A,D)**: FIB-4; **(B,E)**: APRI; **(C,F)**: NFS. FIB-4, Fibrosis-4; APRI, Aspartate Aminotransferase -to-platelet ratio index; NFS, NAFLD fibrosis score.

During the 28 days after randomization, 80.6% (237) patients were discharged and 10.5% (31) died. As shown in Figure 1, the K-M curves showed that patients with FIB-4 >2.67 had a lower hospital discharge and higher death rate during the 28-days (*P* for log-rank test < 0.001) compared to patients with FIB-4 ≤ 2.67. In multivariate analysis, patients with FIB-4 >2.67 were associated with a reduced discharge rate (HR: 0.62, 95% CI: 0.46–0.84) and an increased risk of death (FIB-4 >2.67: HR: 5.13; 95% CI: 2.18–12.07) after full adjustment. When FIB-4 was analyzed as a continuous variable, the results were not statistically significant after full adjustment (28 days discharge: HR: 0.98, 95% CI: 0.95–1.01; 28 days death: HR: 1.02, 95% CI: 1–1.05) (Tables 2, 3).

**Table 2:** The association between Fibrosis scores and 28-days hospital discharge in patients with COVID-19.

**Fibrosis scores**	**Cases (%)**	**Crude**	**Model 1**	**Model 2**	**Model 3**
		**HR (95%CI)**	**HR (95%CI)**	**HR (95%CI)**	**HR (95%CI)**
**FIB-4**
Continuous (*n* = 294)	237 (80.6)	0.95 (0.92, 0.99)^*^	0.95 (0.92, 0.99)^*^	0.96 (0.93, 1.00)^*^	0.98 (0.95, 1.01)
FIB-4 ≤ 2.67 (*n* = 194)	170 (87.6)	Ref	Ref	Ref	Ref
FIB-4 > 2.67 (*n* = 100)	67 (67.0)	0.51 (0.38, 0.68)^*^	0.51 (0.38, 0.68)^*^	0.52 (0.38, 0.69)^*^	0.62 (0.46, 0.84)^*^
**APRI**					
Continuous (*n* = 294)	237 (80.6)	0.90 (0.83, 0.99)^*^	0.90 (0.83, 0.99)^*^	0.91 (0.84, 1.00)^*^	0.96 (0.89, 1.04)
APRI ≤ 1.0 (*n* = 230)	195 (84.8)	Ref	Ref	Ref	Ref
APRI > 1.0 (*n* = 64)	42 (65.6)	0.55 (0.39, 0.76)^*^	0.54 (0.38, 0.75)^*^	0.55 (0.39, 0.77)^*^	0.62 (0.44, 0.87)^*^
**NFS** ^†^					
Continuous (*n* = 262)	210 (80.2)	0.86 (0.80, 0.91)^*^	0.86 (0.80, 0.91)^*^	0.83 (0.78, 0.90)^*^	0.87 (0.81, 0.94)^*^
NFS ≤ 0.676 (*n* = 163)	146 (89.6)	Ref	Ref	Ref	Ref
NFS > 0.676 (*n* = 99)	64 (64.5)	0.46 (0.34, 0.61)^*^	0.46 (0.34, 0.61)^*^	0.41 (0.30, 0.58)^*^	0.50 (0.35, 0.69)^*^

*Model 1 was adjusted for sex*.

*Model 2 was Model 1+ COPD, Diabetes mellitus, coronary artery disease, hypertension, Moderate to severe kidney disease, Congestive heart failure, A prior myocardial infarction, Cerebrovascular disease*.

† *Model 2 was Model 1 + COPD, coronary artery disease, hypertension, Moderate to severe kidney disease, Congestive heart failure, A prior myocardial infarction, Cerebrovascular disease*.

*Model 3 was Model 2+ respiratory SOFA score, Corticosteroids use during hospitalization*.

*BMI, body mass index; COPD, chronic obstructive pulmonary disease; ACEI, angiotension converting enzyme inhibitors; ARB, Angiotensin Receptor Blockers; NSAID, non-steroidal anti-inflammatory drugs; SOFA, Sequential Organ Failure Assessment; BUN, blood urea nitrogen; ALT, alamine aminotransferase; AST, aspartate aminotransferase; ALP, alkaline phosphatase; ALB, albumin; FIB-4, Fibrosis-4; APRI, Aspartate Aminotransferase -to-platelet ratio index*.

* *Represents P < 0.05*.

**Table 3:** The association between Fibrosis scores and 28-days mortality in patients with COVID-19.

**Fibrosis scores**	**Cases (%)**	**Crude**	**Model 1**	**Model 2**	**Model 3**
		**HR (95%CI)**	**HR (95%CI)**	**HR (95%CI)**	**HR (95%CI)**
**FIB-4**
Continuous (*n* = 294)	31 (10.5)	1.03 (1.01, 1.05)^*^	1.03 (1.01, 1.05)^*^	1.03 (1.01, 1.06)^*^	1.02 (1.00, 1.05)
FIB-4 ≤ 2.67 (*n* = 194)	8 (4.1)	Ref	Ref	Ref	Ref
FIB-4 > 2.67 (*n* = 100)	23 (23.0)	6.33 (2.83, 14.17)^*^	6.38 (2.85, 14.27)^*^	5.20 (2.26, 11.93)^*^	5.13 (2.18, 12.07)^*^
**APRI**					
Continuous (*n* = 294)	31 (10.5)	1.12 (1.03, 1.21)^*^	1.13 (1.04, 1.23)^*^	1.11 (1.01, 1.22)^*^	1.08 (0.98, 1.19)
APRI ≤ 1.0 (*n* = 230)	16 (7.0)	Ref	Ref	Ref	Ref
APRI > 1.0 (*n* = 64)	15 (23.4)	3.67 (1.81, 7.43)^*^	3.98 (1.95, 8.11)^*^	3.42 (1.66, 7.06)^*^	2.85 (1.35, 6.03)^*^
**NFS** ^†^
Continuous (*n* = 262)	28 (10.7)	1.41 (1.21, 1.65)^*^	1.39 (1.19, 1.63)^*^	1.40 (1.17, 1.69)^*^	1.28 (1.06, 1.55)^*^
NFS ≤ 0.676 (*n* = 163)	6 (3.7)	Ref	Ref	Ref	Ref
NFS > 0.676 (*n* = 99)	22 (22.2)	6.59 (2.67, 16.25)^*^	6.40 (2.59, 15.80)^*^	5.30 (2.08, 13.49)^*^	4.17 (1.62, 10.72)^*^

*Model 1 was adjusted for sex*.

*Model 2 was Model 1+ COPD, Diabetes mellitus, coronary artery disease, hypertension, Moderate to severe kidney disease, Congestive heart failure*.

† *Model 2 was Model 1+ COPD, coronary artery disease, hypertension, Moderate to severe kidney disease, Congestive heart failure*.

*Model 3. was Model 2+ respiratory SOFA score, Corticosteroids use during hospitalization*.

*BMI, body mass index; COPD, chronic obstructive pulmonary disease; ACEI, angiotension converting enzyme inhibitors; ARB, Angiotensin Receptor Blockers; NSAID, non-steroidal anti-inflammatory drugs; SOFA, Sequential Organ Failure Assessment; BUN, blood urea nitrogen; ALT, alamine aminotransferase; AST, aspartate aminotransferase; ALP, alkaline phosphatase; ALB, albumin; FIB-4, Fibrosis-4; APRI, Aspartate Aminotransferase -to-platelet ratio index*.

* *Represents P < 0.05*.

### Association of APRI Scores With Clinical Outcomes in Patients With COVID-19

Components of APRI scores included AST, platelet scores, ALT, and higher respiratory SOFA scores. Compared to patients with APRI ≤ 1, patients with APRI >1 had lower hospital discharges and higher death rates *(P* for log-rank test < 0.001) (Table 1). In multivariate analysis, patients with APRI >1 had lower hospital discharges (HR: 0.62, 95% CI: 0.44–0.87) and higher deaths (HR: 2.85; 95% CI: 1.35–6.03) through the 28-days after full adjustment. In continuous analysis, there was no significant association between APRI and clinical outcomes after full adjustment (28 days discharge: HR: 0.96, 95% CI: 0.89–1.04; 28 days death: HR: 1.08, 95% CI: 0.98–1.19) (Tables 2, 3).

### Association of NFS Scores With Clinical Outcomes in Patients With COVID-19

Age, BMI, diabetes, AST, ALT, platelet, and albumin were significantly higher in patients with NFS >0.676 compared to patients with NFS ≤ 0.676 (Table 1). Patients with NFS >0.676 were more likely to have chronic health conditions, lower systolic blood pressure, and received more corticosteroids treatment compared to patients with NFS ≤ 0.676.

Among the 262 patients, 210 (80.2%) patients were discharged and 28 (10.7%) died during the 28-days. Compared to patients with NFS ≤ 0.676, patients with NFS >0.676 had a lower hospital discharge and higher death rate (*P* for log-rank test < 0.001). After full adjustment, patients with NFS >0.676 had a lower discharge rate (HR: 0.5, 95% CI: 0.35–0.69) and an increased death rate (HR: 4.17; 95% CI: 1.62–10.72) (Tables 2, 3). When NFS was analyzed as a continuous variable, a positive association was still observed after full adjustment (28 days discharge: HR: 0.87, 95% CI: 0.35–0.69; 28 days death: HR: 1.28, 95% CI: 1.06–1.55) (Tables 2, 3).

### Predictive Ability of ARNI, FIB-4, and NFS Scores in Patients With COVID-19

**Figure 2:**
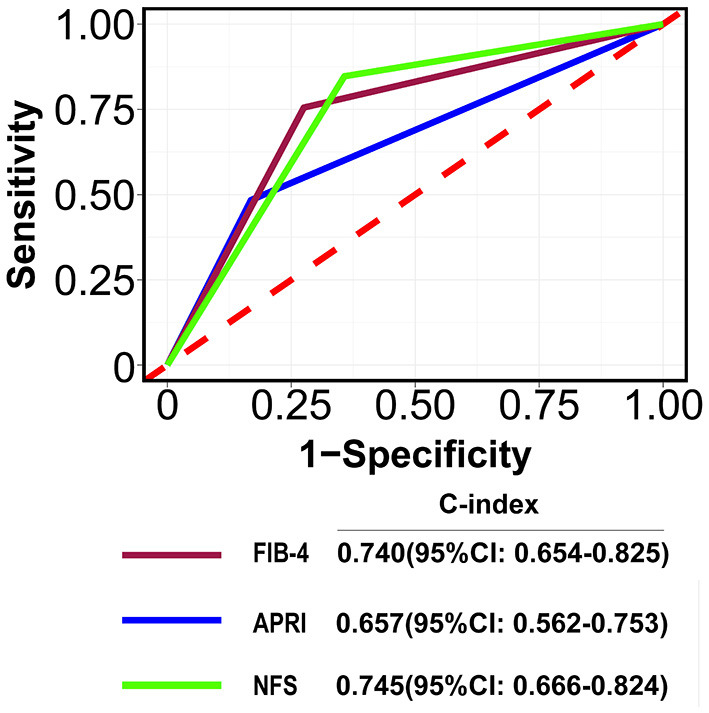
The area under curves for predicting the mortality at 28-days. A: FIB-4; B: APRI; C: NFSFIB-4, Fibrosis-4; APRI, Aspartate Aminotransferase -to-platelet ratio index; NFS: NAFLD fibrosis score.

**Figure 3:**
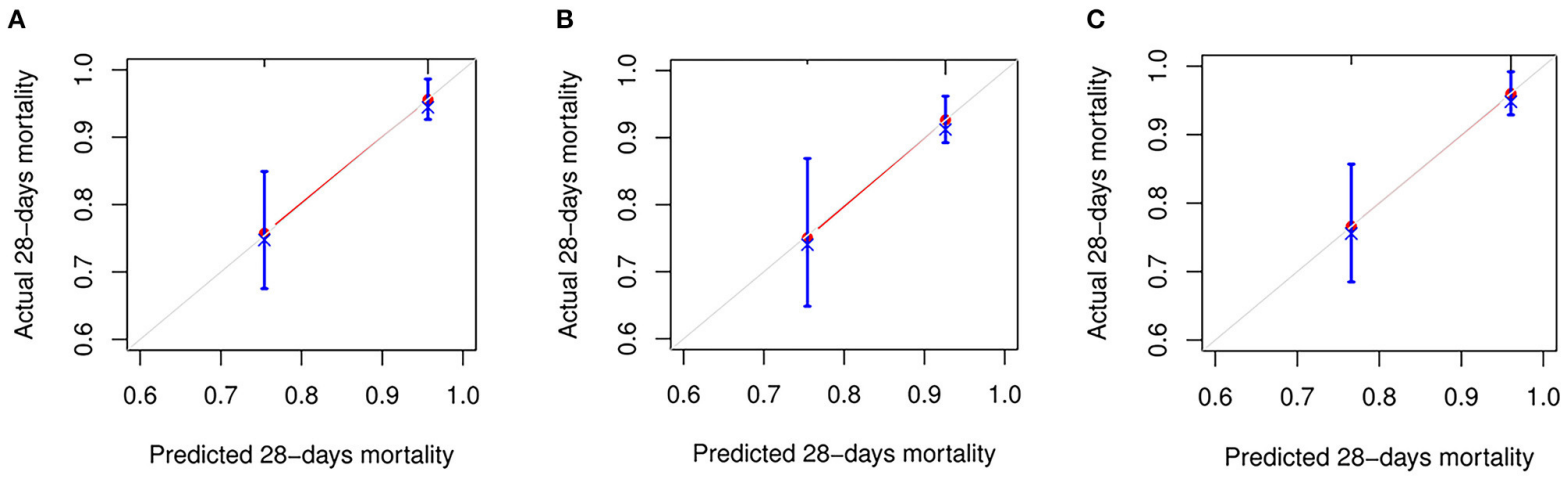
The calibration curve for predicting the mortality at 28-days. **(A)** FIB-4; **(B)** APRI; and **(C)** NFS. FIB-4, Fibrosis-4; APRI, Aspartate Aminotransferase -to-platelet ratio index; NFS, NAFLD fibrosis score.

The C-index for FIB-4, APRI, and NFS scores in predicting 28-days death was 0.74 (95% CI: 0.654–0.825), 0.657 (95% CI: 0.562–0.753), and 0.745 (95% CI: 0.666–0.824), respectively (Figure 2). The performance of FIB-4, APRI, and NFS after internal validation showed a mean C-index of 0.728 (IQR: 0.63–0.829), 0.651(IQR: 0.52–0.783), and 0.72 (0.611–0.833), respectively. Regarding hospital discharge, the C-index for FIB-4, APRI, and NFS scores were 0.649 (95% CI: 0.578–0.719), 0.605 (95% CI: 0.537–0.672), and 0.685 (95% CI: 0.614–0.756), respectively (Supplementary Figure S1). The performance of FIB-4, APRI, and NFS after internal validation showed a mean C-index of 0.649 (IQR: 0.573–0.72), 0.606 (IQR: 0.534–0.667), and 0.688 (IQR: 0.61–0.761), respectively. The calibration curve showed a good agreement between predicted and observed outcomes among all liver fibrosis scores (Figure 3 and Supplementary Figure S2).

### Supplement Analysis and Sensitive Analysis

For 28-day death, ECMO or death, or for in-hospital death, higher APRI, FIB-4, and NFS scores were associated with adverse outcomes (all *P* < 0.05) (Supplementary Tables S3, S4). The association between FIB-4, APRI, and NFS was still significant (*P* < 0.05, data not shown) in sensitive analysis that excluded patients with chronic liver disease or when a competing model was used.

## Discussion

Our *post-hoc* analysis of the ORCHID trial suggested the following results: First, fibrosis scores, including FIB-4 scores, APRI scores, and NFS scores, were significantly associated with a lower discharge rate and an increased death rate in hospitalized patients with COVID-19. Second, all three fibrosis scores had a moderate discriminatory ability for predicting death or discharge during the 28 days. To our best of knowledge, this is the first study that compressively assessed the fibrosis scores and clinical outcomes of patients with COVID-19.

Previous studies have demonstrated that pre-existing chronic liver diseases were an independent predictor of adverse outcomes in patients with COVID-19 (14, 15). In our analysis, we found that non-invasive liver fibrosis scores were positively correlated with adverse outcomes in patients with COVID-19. The proportion of patients with chronic liver disease was 2.4%. However, advanced liver disease ranged from 22 to 37%, which were assessed through liver fibrosis scores. This result was consistent with the view that underlying chronic liver disease may be underestimated in patients infected with SARS-CoV-2. Supporting this, the study of Sterling et al. (16) demonstrated that although the prevalence of known underlying liver diseases was 6%, there was a high percentage (52%) of patients with FIB-4 >2.67. When patients with chronic liver disease were excluded from our analysis, the positive association of liver fibrosis scores and worse prognosis with FIB-4 was still observed in our sensitivity analysis. These results highlighted the value of screening liver fibrosis in patients with COVID-19, even in patients without chronic liver disease. Overall, liver fibrosis scores may be a novel and promising prognostic marker for predicting adverse outcomes in patients with the COVID-19.

However, these non-invasive assessments should be interpreted with caution due to complexities arising from COVID-19 progression. Liver injury is one of the most common complications in patients with COVID-19 (2). Studies have shown that indicators for liver injury (i.e., AST and ALT) are significantly increased during COVID-19 and are important predictors for all-cause death (15). FIB-4 levels have been shown to correlate with SARS-CoV-2 plasma RNA levels and monocyte-associated cytokine levels (17). In addition to the underlying prevalence of chronic liver diseases, patients with higher liver fibrosis scores have been linked with COVID-19 disease pathogenesis and severity (18, 19).

### Comparison With the Previous Studies

Previous studies have reported the association between different categories of FIB-4 and outcomes in patients with COVID-19 Supplementary Table S5). Elfeki et al. (20) and Samaniego et al. demonstrated that FIB-4 scores of 1–2.67 were not associated with an increased risk of death. However, Calapod et al. (21) found that moderate FIB-4 scores (1.3–2.67) could predict severe COVID-19. Xiang et al. (22) reported that FIB-4 >1.45 was associated with an increased risk of severe COVID-19. In our analysis, we used a cutoff of liver fibrosis (which was defined as advanced liver fibrosis) and did not investigate the predictive ability of FIB-4, APRI, or NFS for moderate liver fibrosis. Additional studies should be performed to investigate their predictive ability. Sterling et al. assessed the predicted ability of FIB-4, known respiratory disease, cardiac disease, liver disease, diabetes mellitus, and obesity on MV, with a C-index of 0.79 (16). In this study, we found all fibrosis scores had the good predictive ability and calibration performance for death. Among them, NFS and FIB-4 appeared to have better performance compared to APRI. However, to date, only a limited number of studies have reported C-indices for FIB-4, APRI, or NFS.

Previous studies have demonstrated that AST and ALT were significantly associated with death in patients with COVID-19 (2, 23). In our study, for all components encompassing the liver fibrosis score, there was no significant association between ALT, platelet, or clinical outcomes. The difference may be due to patient heterogeneity. A combination in the liver fibrosis scores but not their components may help predict clinical outcomes in patients with COVID-19.

### Clinical Implications

Our results showed that liver fibrosis scores were associated with poor prognosis and maybe a simple marker for predicting severity and death in patients with COVID-19. All components of these liver fibrosis indices (e.g., age, AST, and ALT) were simple and inexpensive to determine. However, our study does not support performing a liver biopsy, which is the current gold standard for assessing liver fibrosis, to predict adverse outcomes in patients with COVID-19. Performing a liver biopsy may be challenging and not suitable for patients. As mentioned previously, these non-inversive liver fibrosis scores may be the result of complex factors involved in the progression of COVID-19 and should not be considered as an assessment for liver fibrosis.

### Strengths and Limitations

This is the first study that comprehensively assessed the association of liver fibrosis scores, including APRI and NFS scores, with adverse outcomes in patients with COVID-19. However, there were several limitations to this study. First, this was a *post-hoc* analysis from RCTs; hence, the intrinsic limitations of observational study preclude us from drawing a causal link. Measure and unmeasured confounding factors may have influenced our results. The prospective design would have reduced the possibility of reverse causality, while a multi-center design would have reduced selection bias. Second, previous clinical laboratory data were not available for the majority of patients. Hence, we are unsure whether higher liver enzymes were due to COVID-19 or comorbidities due to existing liver disease. Third, our dataset does not collect information on variants of COVID-19. Finally, our analysis was performed on a moderately-sized sample cohort. Larger patient cohorts are needed to validate our findings.

## Conclusion

Our *post-hoc* analysis of patients in the ORCHID trial demonstrated that liver fibrosis scores, including FIB-4, APRI, and NFS were significantly associated with reduced hospital discharge rates and higher risk of all-cause death.

## Data Availability Statement

The original contributions presented in the study are included in the article/Supplementary Material, further inquiries can be directed to the corresponding author/s.

## Ethics Statement

The studies involving human participants were reviewed and approved by Central Institutional Review Board at Vanderbilt University Medical Center. The patients/participants provided their written informed consent to participate in this study.

## Author Contributions

PY and XL were responsible for the entire project and revised the draft. JZ, FL, ZL, PX, XT, and MX performed the data extraction, statistical analysis, interpreted the data, and drafted the first version of the manuscript. TS, YS, and JM revised the manuscript. All authors participated in the interpretation of the results and prepared the final version of the manuscript.

## Funding

This work was supported by the grant from the Natural Science Foundation in Jiangxi Province grant (No. 202002BAB216022 to JZ and Nos. 20192ACBL21037 and 202004BCJL23049 to PY) and the National Natural Science Foundation of China (No. 82160371 to JZ, No. 82100869 to PY, and No. 21866019 to JM).

## Conflict of Interest

The authors declare that the research was conducted in the absence of any commercial or financial relationships that could be construed as a potential conflict of interest.

## Publisher's Note

All claims expressed in this article are solely those of the authors and do not necessarily represent those of their affiliated organizations, or those of the publisher, the editors and the reviewers. Any product that may be evaluated in this article, or claim that may be made by its manufacturer, is not guaranteed or endorsed by the publisher.
