# Supplementary material for: Liver Fibrosis Scores and Clinical Outcomes in Patients With COVID-19
Source: Front Med (Lausanne). 2022 Apr 8;9:829423. doi: 10.3389/fmed.2022.829423 (PMC9024307; doi:10.3389/fmed.2022.829423)
Supplement: Supplementary file 1 [file Data_Sheet_1.pdf]

Supplemental materials

Liver fibrosis scores and clinical outcomes in COVID-19 patients

Table S1. The Univariate Cox regression analyses of the components of FIB-4, APRI and NFS with 28-days hospital discharge, mortality in patients with COVID-19

	Hospital discharge	Mortality
	HR (95% CI)	HR (95% CI)
**FIB-4, APRI(N=294)**		
Age, 10 years	0.92(0.91,0.93)*	1.37(1.30,1.44)*
ALT, 10U/L	1.000(0.998,1.002)	1.004(1.002,1.007)*
AST, 10U/L	0.996(0.992,0.999)*	1.009(1.006,1.011)*
Platelet, 10/mm3	1.008(1.007,1.010)*	0.983(0.976,0.990)*
**NFS(N=262)**		
Diabetes	0.86(0.65,1.14)	1.92(0.91,4.02)
BMI, kg/m2	1.00(0.98,1.01)	0.99(0.96,1.03)
Age, 10 years	0.92(0.91,0.94)*	1.37(1.30,1.45)*
ALT, 10U/L	1.000(0.997,1.003)	1.009(1.006,1.011)*
AST, 10U/L	0.987(0.981,0.993)*	1.008(1.006,1.011)*
ALB, g/dL	1.37(1.16,1.62)*	0.51(0.30,0.86)*
Platelet, 10/mm3	1.008(1.006,1.011)*	0.987(0.979,0..995)*

Abbreviation: Fibrosis-4: FIB-4, Aspartate Aminotransferase -to-platelet ratio index: APRI; Non-alcoholic fatty liver disease fibrosis: NFS; ALT: alamine aminotransferase; AST: aspartate aminotransferase; ALB: albumin

*P<0.05

Table S2. The association between Fibrosis scores and ECMO or Death in patients with COVID-19

Fibrosis scores	Cases (%)	Crude HR (95% CI)	Model 1 HR (95% CI)	Model 2 HR (95% CI)	Model 3 HR (95% CI)
FIB-4					
Continuous (n=294)	37(12.6)	1.02(1.00,1.05)*	1.02(1.00,1.05)*	1.03(1.00,1.05)*	1.01(0.99,1.04)
FIB-4≤2.67(n=194)	10(5.2%)	Ref	Ref	Ref	Ref
FIB-4>2.67(n=100)	27(27.0%)	5.93(2.87,12.25)*	5.95(2.87,12.27)*	5.40(2.56,11.39)*	4.95(2.30,10.66)*
APRI					
Continuous (n=294)	37(12.6)	1.08(0.99,1.18)	1.09(1.00,1.20)	1.08(0.98,1.19)	1.02(0.92,1.13)
APRI≤1.0(n=230)	18(7.8%)	Ref	Ref	Ref	Ref
APRI>1.0(n=64)	19(29.7%)	4.22(2.21,8.04)*	4.51(2.35,8.66)*	4.17(2.16,8.06)*	3.29(1.65,6.55)*
NFS†					
Continuous (n=262)	34(13.0)	1.43(1.25,1.64)*	1.43(1.24,1.65)*	1.47(1.25,1.72)*	1.34(1.14,1.58)*
NFS≤0.676(n=163)	6(3.7%)	Ref	Ref	Ref	Ref
NFS>0.676(n=99)	28(28.3%)	8.73(3.61,21.09)*	8.57(3.54,20.72)*	8.17(3.32,20.12)*	6.41(2.59,15.87)*

Model 1 adjusted for sex;

Model 2: Model 1+ COPD, Diabetes mellitus, coronary artery disease, hypertension, moderate to severe kidney disease, congestive heart failure;

†Model 2: Model 1+ COPD, coronary artery disease, hypertension, moderate to severe kidney disease, congestive heart failure;

Model 3: Model 2+ respiratory SOFA score, Corticosteroids use during hospitalization;

Abbreviations: COPD, chronic obstructive pulmonary disease; SOFA, Sequential Organ Failure Assessment; BUN, blood urea nitrogen; FIB-4, Fibrosis-4; APRI, Aspartate Aminotransferase -to-platelet ratio index; ECMO, extracorporeal membrane oxygenation

Table S3. The association between Fibrosis scores and hospital mortality in patients with COVID-19

Fibrosis scores	Cases (%)	Crude HR (95% CI)	Model 1 HR (95% CI)	Model 2 HR (95% CI)	Model 3 HR (95% CI)
FIB-4					
Continuous (n=294)	34(11.6)	1.03(1.01,1.05)*	1.03(1.01,1.05)*	1.03(1.01,1.06)*	1.02(1.00,1.05)
FIB-4≤2.67(n=194)	10(5.2%)	Ref	Ref	Ref	Ref
FIB-4>2.67(n=100)	24(24.0%)	5.29(2.53,11.08)*	5.32(2.54,11.13)*	4.53(2.12,9.70)*	4.35(1.98,9.56)*
APRI					
Continuous (n=294)	34(11.6)	1.11(1.03,1.21)*	1.12(1.04,1.22)*	1.11(1.01,1.21)*	1.08(0.98,1.19)
APRI≤1.0(n=230)	18(7.8%)	Ref	Ref	Ref	Ref
APRI>1.0(n=64)	16(25.0%)	3.48(1.77,6.83)*	3.75(1.90,7.42)*	3.28(1.64,6.55)*	2.69(1.31,5.49)*
NFS					
Continuous (n=262)	31(11.8)	1.36(1.17,1.57)*	1.34(1.15,1.56)*	1.35(1.13,1.61)*	1.22(1.02,1.46)*
NFS≤0.676(n=163)	7(4.3%)	Ref	Ref	Ref	Ref
NFS>0.676(n=99)	24(24.2%)	6.15(2.65,14.27)*	6.00(2.58,13.94)*	5.28(2.21,12.63)*	3.94(1.63,9.55)*

Model 1 was adjusted for sex;

Model 2 was Model 1+ COPD, Diabetes mellitus, coronary artery disease, hypertension, moderate to severe kidney disease, congestive heart failure;

†Model 2 was Model 1+ COPD, coronary artery disease, hypertension, moderate to severe kidney disease, congestive heart failure;

Model 3. was Model 2+ respiratory SOFA score, Corticosteroids use during hospitalization;

Abbreviations: COPD, chronic obstructive pulmonary disease; SOFA, Sequential Organ Failure Assessment; BUN, blood urea nitrogen; FIB-4, Fibrosis-4; APRI, Aspartate Aminotransferase -to-platelet ratio

Table S4. The association between Fibrosis scores and outcomes in patients with COVID-19 and without liver disease.

Fibrosis scores		Hospital discharge† HR (95% CI)	Mortality‡ HR (95% CI)
FIB-4			
	Continuous	0.98(0.95,1.01)	1.03(1.00,1.05)
	FIB-4≤2.67	Ref	Ref
	FIB-4>2.67	0.61(0.45,0.83)*	5.25(2.24,12.34)*
APRI			
	Continuous	0.96(0.89,1.04)	1.08(0.98,1.19)
	APRI≤1.0	Ref	Ref
	APRI>1.0	0.62(0.44,0.87)*	2.91(1.38,6.12)*
NFS§			
	Continuous	0.87(0.80,0.93)*	1.31(1.08,1.59)*
	NFS≤0.676	Ref	Ref
	NFS>0.676	0.48(0.34,0.67)*	4.34(1.69,11.19)*

†Fibrosis scores was adjusted sex, COPD, Diabetes mellitus, coronary artery disease, hypertension, moderate to severe kidney disease, congestive heart failure, A prior myocardial infarction, Cerebrovascular disease, respiratory SOFA score and Corticosteroids use during hospitalization;

†§Fibrosis scores was adjusted sex, COPD, coronary artery disease, hypertension, moderate to severe kidney disease, congestive heart failure, A prior myocardial infarction, Cerebrovascular disease, respiratory SOFA score and Corticosteroids use during hospitalization;

‡Fibrosis scores was adjusted sex, COPD, Diabetes mellitus, coronary artery disease, hypertension, moderate to severe kidney disease, congestive heart failure, respiratory SOFA score and Corticosteroids use during hospitalization;

‡§Fibrosis scores was adjusted sex, COPD, coronary artery disease, hypertension, moderate to severe kidney disease, congestive heart failure, respiratory SOFA score and Corticosteroids use during hospitalization;

Abbreviations: COPD, chronic obstructive pulmonary disease; SOFA, Sequential Organ Failure Assessment; BUN, blood urea nitrogen; FIB-4, Fibrosis-4; APRI, Aspartate Aminotransferase -to-platelet ratio

Table S5. A summary of studies for the association between liver scores and clinical outcomes in patients with COVID-19

Author, publication year, country	Study design	Sample size	Liver fibrosis score reported	Major findings
Xiang(1), 2020, China	Retrospective cohort	267	FIB-4<br>< 1.45<br>1.45-3.25<br>> 3.25	FIB-4>3.25 was associated with 12-fold (HR:12.47, 95% CI: 2.3-68.7) increased risk of high-flow oxygen use and 11-fold (OR: 11.92, 95% CI: 3.1-45) increased risk of progress to severe disease after adjusted for sex, hypertension, DM, heart diseases, liver diseases, kidney diseases, psychological disorder, time from admission to symptom onset date, D-dimer, CRP
Elfeki(2), 2021, USA	Retrospective cohort	373	FIB-4<br>< 1.30<br>1.30–2.67<br>> 2.67	Among patients at risk of NAFLD with COVID-19 infection, Patients with FIB-4 index (1.30–2.67) had 67% higher risk of hospitalization (OR: 1.67, 95% CI: 1.06–2.64) and patients with high FIB-4 index (>2.67) had higher risk of mortality OR 2.22 (95% CI: 1.20–4.12) compared to low category with an after adjusted for Type 2 DM, and CKD
Ibáñez-Samaniego(3), 2021, Spain	Retrospective cohort	160	FIB-4<br>< 2.67<br>≥ 2.67	FIB-4 ≥2.67 (OR: 3.41, 95%CI: 1.30–8.92) increased significantly the risk of ICU admission after adjusted for Hypertension, respiratory disease,

				and bilirubin, LDH acute C-reactive protein.
Calapod(4), 2020, Romania	Prospective cohort	138	FIB-4<br>< 1.30<br>1.30–2.67<br>> 2.67	FIB-4 ≥3.25 (OR:4.89, 95% CI: 1.34–12.3) was associated with increased risk for developing severe COVID-19 after adjusted for sex, BMI, dyspnea, ferritin, CRP, AST, and ALT
Sterlin(5),2020, USA	Retrospective cohort	256	FIB-4<br>< 2.67<br>≥ 2.67	FIB-4 ≥2.67 was also associated with increased 30-day mortality (OR: 8.4, 95% CI: 2.23–31.7). Model including Fibrosis-4 Index ≥2.67, known respiratory disease, cardiac disease, liver disease, diabetes mellitus, and obesity had an area under the receiver operating characteristic curve of 0.79.

Figure S1.

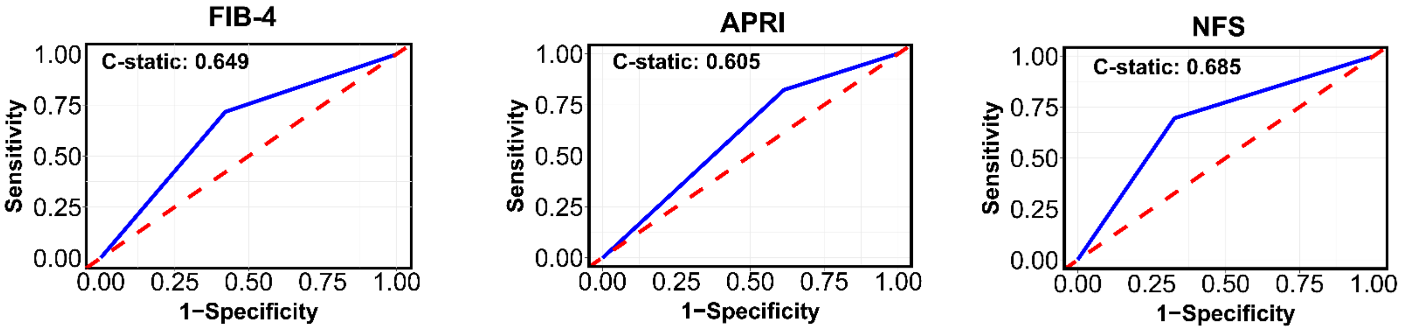

Figure S1: the area under curves for predicting the hospital discharge at 28-days A: FIB-4; B: APRI; C: NFS

Figure S2.

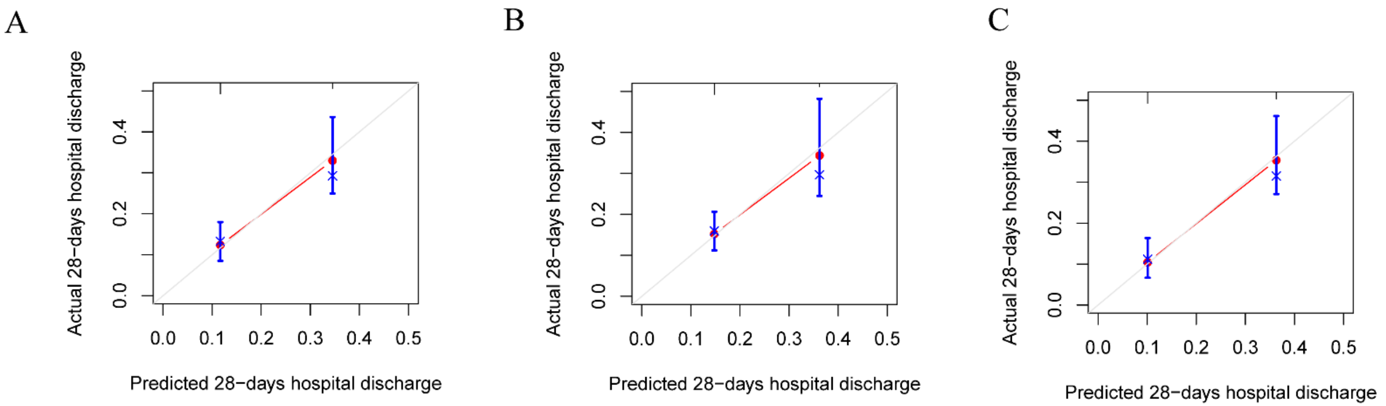

Figure S2: the calibration curve for predicting the hospital discharge at 28-days A: FIB-4; B: APRI; C: NFS

1. Xiang F, Sun J, Chen P-H, Han P, Zheng H, Cai S, Kirk GD. Early elevation of FIB-4 liver fibrosis score is associated with adverse outcomes among patients with COVID-19. medRxiv. 2020.

2. Elfeki MA, Robles J, Akhtar Z, Ullah F, Ganapathiraju I, Tran C, Inman C, Collin SM, Rosa R. Impact of Fibrosis-4 Index Prior to COVID-19 on Outcomes in Patients at Risk of Non-alcoholic Fatty Liver Disease. Dig Dis Sci. 2021 Jun 26. doi:10.1007/s10620-021-07120-0. Cited in: Pubmed; PMID 34173917.

3. Ibanez-Samaniego L, Bighelli F, Uson C, Caravaca C, Fernandez Carrillo C, Romero M, Barreales M, Perello C, Madejon A, Marcos AC, Albillos A, Fernandez I, Garcia-Samaniego J, Calleja JL, Banares R. Elevation of Liver Fibrosis Index FIB-4 Is Associated With Poor Clinical Outcomes in Patients With COVID-19. J Infect Dis. 2020 Aug 4;222(5):726-733. doi:10.1093/infdis/jiaa355. Cited in: Pubmed; PMID 32563190.

4. Calapod OP, Marin AM, Onisai M, Tribus LC, Pop CS, Fierbinteanu-Braticevici C. The Impact of Increased Fib-4 Score in Patients with Type II Diabetes Mellitus on Covid-19 Disease Prognosis. Medicina (Kaunas). 2021 Apr 30;57(5). doi:10.3390/medicina57050434. Cited in: Pubmed; PMID 33946377.

5. Sterling RK, Oakes T, Gal TS, Stevens MP, deWit M, Sanyal AJ. The Fibrosis-4 Index Is Associated With Need for Mechanical Ventilation and 30-Day Mortality in Patients Admitted With Coronavirus Disease 2019. J Infect Dis. 2020 Nov 9;222(11):1794-1797. doi:10.1093/infdis/jiaa550. Cited in: Pubmed; PMID 32856702.
